# “Out Like a Lion:” Terminating the COVID-19 National Public Health Emergency

**DOI:** 10.1017/jme.2023.71

**Published:** 2023

**Authors:** James G. Hodge

**Affiliations:** 1. ARIZONA STATE UNIVERSITY, PHOENIX, AZ, USA

**Keywords:** COVID-19, Pandemic, Public Health Emergency, Termination, Impacts, Immigration

## Abstract

From its inception, the COVID-19 pandemic has been a disruptive force on U.S. health care and public health systems. President Biden’s announced termination of the national public health emergency on May 11, 2023 portends a return to normalcy and relief for Americans from the greatest infectious disease scourge the nation has ever faced. In reality, closing out this pandemic presents a tempest of legal and practical complications.

Following persistent rumors and repeated Congressional calls for action since mid-2021,^1^ President Biden formally announced on January 30, 2023 that national declarations of emergency for the COVID-19 pandemic would officially end on May 11, 2023.^2^ Ideally the pandemic which has claimed over 350,000 Americans in each of the last three years between 2020-2022 would go “out like a lamb” with the termination of the national public health emergency (PHE). Yet, closing out the emergencies underlying the single greatest infectious disease threat to ever confront the United States is not easy. As explained below, it seems COVID-19 is destined to go out just as it came in: “like a lion.”

Terminating the national PHE carries severe repercussions for national health care and public health systems. Millions stand to lose health insurance coverage. Millions more await treatments for non-COVID conditions put aside during the pandemic. Unpredictable levels of care for “long-COVID” and associated mental health harms from months of social isolation and disruption add to the challenge. A health care system already battered by repeated waves of COVID-19 infections also faces other emerging disease threats (e.g., RSV, annual flu, measles) and massive rises in morbidity and “deaths of despair” from illicit drugs. The national PHE in response to America’s second worst epidemic, opioid misuses, has already outlasted the COVID-19 pandemic emergency by over three years.^3^

Public health systems stand to lose as well. State and local public health agencies have been besieged by significant efforts in largely conservative states to curtail their emergency^4^ and routine public health powers.^5^ National public health surveillance and readiness hinge on sustained funding. Congress, however, seems committed to defunding public health services, refusing to provide even base-level resources to continue surveillance, testing, and vaccination efforts essential to quell new strains.^6^ Consequently, COVID-19 variants may continue to plague the nation and claim American lives for years ahead much like annual influenza.

Capping these impacts are profound immigration disputes. For months, President Biden and the Centers for Disease Control and Prevention (CDC) have sought an end to restrictive border control measures initially instituted by the Trump Administration in response to COVID-19. Standing in the way have been both Republicans and Democrats opposed to the removal and re-institution, respectively, of immigration limits cloaked as public health protections.^7^ Divergent litigation snaked its way to the U.S. Supreme Court in 2022. Ending the national PHE could have helped settle legal controversies. In reality, it may only exacerbate them further, implicating the lives and safety of hundreds of thousands of persons.

**“When,” Not “If.”** From the moment the COVID-19 pandemic was classified as a national PHE by former Health and Human Services (HHS) Secretary Alex Azar on January 31, 2020,^8^ it was destined to end. Following ten consecutive 90-day renewals via HHS,^9^ the only question became “when.” After President Biden inappropriately suggested that the “pandemic is over” on September 18, 2022, immediate calls for rescissions of national and state declarations of emergency arose. Most states have already withdrawn their emergency declarations. As of February 1, 2023, the National Governors Association reported that only nine states retain their original COVID-19 states of emergency.^10^ When the President finally announced the end of the national emergency set for May 11, 2023, de-escalating legal support for emergency response efforts began. Federal agencies like HHS, CDC, the Food and Drug Administration (FDA), and Centers for Medicare and Medicaid Services (CMS) undertook immediate efforts to help transition the nation’s health care and public health systems back to levels of normalcy. States still under their own emergency declarations announced plans to rescind their statuses in sync with the federal termination.

Yet, not all federal emergency declarations tied to the COVID-19 pandemic are projected to end. HHS’ distinct declarations under the Public Readiness and Emergency Preparedness (PREP) Act^11^ authorize emergency use and implementation of medical countermeasures, including COVID-19 tests and vaccinations not otherwise fully approved by FDA.^12^ The untimely withdrawal of PREP Act authorities, including liability protections for vaccine manufacturers and preemptive measures negating conflicting state laws,^13^ could be disastrous.^14^ Consequently PREP Act declarations and amendments extending from the pandemic are anticipated (but not guaranteed) to remain in place through at least 2024.^15^

Even as most Americans see COVID-19 in the nation’s rear-view mirror and are diametrically opposed to new rounds of preventative measures, epidemiologists are concerned that the pandemic is not winding down sufficiently.^16^ New infectious strains of coronavirus are emerging — yet again. For the week of February 8-15, CDC reported 260,000 new infections nationally.^17^ Since the start of 2023, the U.S. has averaged nearly 495 COVID-19 deaths per day.^18^ While these numbers represent precipitous declines from the height of the pandemic, if mortality trends continue, an additional 180,000 Americans will be lost to COVID-19 in 2023, establishing it as the fifth leading cause of death nationally.^19^ Sadly, most of these COVID-19 deaths are preventable if only more Americans were fully vaccinated and observed modest public health recommendations.

Whether President Biden’s immigration stances ever take effect is indeterminate under Supreme Court adjudications that have disfavored highly-restrictive immigration policies. Just as pandemic mismanagement contributed to President Trump’s re-election loss in 2020, however, President Biden may face his own reckoning in 2024 from the nation’s “broken immigration system”and resulting crisis at the border.

**Managing Health Care and Public Health Impacts.** HHS’ PHE authorizes extensive legal options to buttress the nation’s public health and health care systems in crises. Coupled with presidential emergency declarations and prior congressional actions, the national PHE facilitates substantial health care and public health response efforts and real-time shifts to crisis standards of care.^20^ These essential emergency services, however, may dissipate when the PHE concludes. The ramifications are immense.

More than 15 million Americans may lose their temporary health coverage under Medicaid and Children’s Health Insurance Programs by the end of 2023 without additional federal intervention.^21^ Health care service innovations including telehealth initiatives^22^ and health care worker accommodations via licensure reciprocity, scope of practice, and liability protections,^23^ may be curtailed within months.^24^ Responding to patient surges without these legal options in future COVID-19 outbreaks will be problematic.

FDA’s authorities to fast-track authorization of drugs, vaccines, tests, and protective equipment rely in large part on legal foundations of afore-mentioned PREP Act authorities. While these authorities are not immediately on the “chopping block,” PREP Act declarations rely on substantiated emergency circumstances typically warranting a national PHE. In essence, the national PHE largely validates PREP Act protections. Terminating the PHE may ultimately diminish FDA’s emergency use authorities for a range of medical countermeasures if challenged in court.^25^

Public health surveillance efforts to monitor COVID-19 cases are already being phased out due to insubstantial funding, which lends to risks of new COVID-19 variants. Specific public health interventions to test, screen, vaccinate, trace, and investigate COVID-19 cases all rely in part on the national PHE. The collective rescission of these health services and public health programs presents its own crisis, especially for exhausted health care workers and public health officials chastised for their actions and failures during the pandemic itself. As the White House, HHS, and other federal authorities prepare public and private health actors for renewed roles post-emergency,^26^ they must also consider how to mend battered health systems facing an onslaught of continued population health challenges.^27^

**Immigration Controversies at the Border.** The premise that ending the national PHE on May 11, 2023 will bring closure to raging immigration legal battles on the U.S. southern border is specious. On March 20, 2020 CDC issued an order^28^ restricting border immigration via the Public Health Services Act.^29^ Known as a “Title 42” order, it essentially allowed federal agents to reject persons at the border, even those seeking asylum, on grounds they present risks of transmitting infectious diseases. Early on in the pandemic, those risks seemed arguably plausible. Only months later did reports surface that the Trump administration forced CDC to issue the order against its scientists’ assessments.^30^ Still, even after presidential administrations changed hands, CDC re-issued the same order on August 2, 2021, with President Biden’s acquiescence.^31^ To date, upwards of two million persons seeking entry into the U.S. have been turned away over the course of the pandemic.^32^

When CDC finally pronounced its plan to terminate its Title 42 order by May 23, 2022,^33^ immediate legal objections arose. Nearly half the states sued to keep the order in place. A Louisiana federal district court blocked CDC’s attempt to lift it three days prior to its rescission.^34^ In separate litigation brought by asylum-seeking families in the District of Columbia, a federal court ruled inappositely on November 15, 2022, essentially requiring CDC to pull its order once and for all.^35^ Led by Arizona, multiple states’ attorneys generals filed an emergency application before the U.S. Supreme Court in *Arizona v. Mayorkas* ^36^ to stay the D.C. district court ruling. The Supreme Court agreed to review the case and granted the states’ temporary request,^37^ essentially keeping CDC’s original Title 42 order in place.

The plot thickened from there. When President Biden announced the end of the national PHE, he also opined that its rescission would affirmatively close out CDC’s Title 42 order.^38^ The legal premise was simple enough. Since CDC stated in the initial order that it was relying on the national PHE as authority for its issuance, withdrawing the PHE logically meant an end of the Title 42 order. It is a plausible argument although CDC actually has independent legal authority under the Public Health Service Act to issue Title 42 orders. Still, the Supreme Court apparently agreed with the Biden Administration, removing the *Mayorkas* case from its docket on February 17, 2023,^39^ notwithstanding objections from several Republican-controlled states. CDC’s Title 42 order seems destined to end in mid-May after over three years of continuances.

Immigration policies replacing it, however, may actually be worse. On January 5, 2023, the Administration, perhaps sensing political trouble ahead of an election year,^40^ proposed a new order restricting asylum-seekers.^41^ President Biden’s temporary order, due to take effect just prior to the termination of Title 42, would require many persons to seek asylum in other countries before they may be considered for asylum in the U.S.^42^ Later, on March 6, the Administration intimated it may temporarily detain asylum-seekers, including their children, crossing the border.^43^ Some Congressional Democrats assimilated the proposals to President Trump’s anti-immigration policies and an affront to international human rights.^44^ Whether President Biden’s immigration stances ever take effect is indeterminate under Supreme Court adjudications that have disfavored highly-restrictive immigration policies. Just as pandemic mismanagement contributed to President Trump’s re-election loss in 2020, however, President Biden may face his own reckoning in 2024 from the nation’s “broken immigration system”^45^ and resulting crisis at the border.
